# Staphylococcal Enterotoxin B (SEB) Induces Memory CD4 T Cell Anergy *in vivo* and Impairs Recall Immunity to Unrelated Antigens

**DOI:** 10.4172/2155-9899.1000346

**Published:** 2015-07

**Authors:** David K Janik, William T Lee

**Affiliations:** 1 The Department of Biomedical Sciences, School of Public Health, The University at Albany, USA; 2 The Laboratory of Immunology, The Wadsworth Center, New York State Department of Health, USA

**Keywords:** Immunological memory, Recall immunity, Clonal anergy, Superantigens, T Lymphocytes, Immune tolerance, Helper T cells

## Abstract

**Introduction:**

Naïve and memory T cells can utilize unique regulatory pathways to promote protection but prevent self-reactivity. A bacterial superantigen SEB exploits unique TCR proximal signaling processes in memory CD4 T cells to induce clonal anergy. The aim of this study was to determine if SEB could antagonize memory CD4 T cells *in vivo* and whether there would be consequences on recall immune responses. We evaluated Ab responses to a T-dependent antigen as a measurement of memory T cell helper function.

**Method:**

BALB/c mice were primed with TNP-RGG to elicit memory B cells and also immunized with an ovalbumin peptide to elicit memory helper T cells. Another group of TNP-RGG immunized mice were used as adoptive transfer recipients of exogenous DO11.10 memory T cells. Mice were challenged with TNP-OVA with or without prior administration of SEB. B cells secreting IgM or IgG TNP-specific Ab were enumerated by ELISPOT as indicators of primary versus secondary humoral immunity.

**Results:**

Comparing the SEB and non-SEB-treated groups, the SEB-treated group failed to produce TNP-specific IgG in response to challenge with TNP-OVA, even if they were previously immunized with OVA. All groups produced IgM, indicating that the primary Ab responses and naïve helper T cells were not impacted by SEB. SEB had no negative impact when DO11.10 × Fyn^−/−^ memory T cells were used as donor cells.

**Conclusion:**

The present study indicated that SEB selectively targeted memory CD4 T cells *in vivo* and prevented helper function. Consequently, recall humoral immunity was lost. The data are most consistent with *in vivo* T cell anergy as opposed to indirect suppression as elimination of Fyn kinase restored helper function. These data suggest that bacterial superantigens can impair post-vaccination memory cell responses to unrelated antigens via their ability to target Vb families and antagonize memory cell activation.

## Introduction

An initial encounter with foreign antigen stimulates T lymphocyte proliferation and differentiation into “antigen experienced” effector and memory cells. During subsequent exposures to the same antigen, experienced T cells respond more rapidly and vigorously [1–5]. While the frequency of antigen-specific memory lymphocytes may be elevated as compared to naïve cells, a portion of the robust memory response can also be attributed to specific features of experienced cells. Unlike naïve cells, effector and memory T cells rapidly secrete a broad array of lymphokines. Further, memory cells may be activated more quickly due to altered requirements for costimulation, increased adhesion marker expression and, possibly, an increased sensitivity of signaling through the antigen receptor [6,7]. Like naïve cells, improper responses against self-antigens by memory cells must be prevented. Because of easier activation, it is likely that memory cells utilize additional or different regulatory mechanisms to prevent inappropriate activation.

Stimulation of T cells by peptide-bearing APCs, involves multiple signal transduction pathways. Several studies have shown that signaling through the TCR itself is influenced by the differentiation state of the cells or the specific stimulus (e.g. self-antigen/foreign peptide, superantigen, anti-TCR Abs) and, additionally, that the functional outcome might differ (e.g. activation or tolerance). For example, there is *in vitro* and *in vivo* evidence for T cell antagonism by altered peptide ligands that differ from canonical ligands in only a single or a few amino acids [8]. Previous studies have shown that T cell antagonism *in vitro* is accompanied by differential signal transduction [9]. Likewise, naïve and memory T cells may respond to the same stimulus differently. For example, soluble, but not plate-bound TCR/CD3-specific antibodies [10,11], and superantigens [12] stimulate proliferation by naive CD4 T cells but not by memory CD4 T cells. Again, TCR-mediated signaling is different in the two cell types in response to the different stimuli [13,14].

Pathogens cause disease and subvert host defense mechanisms using a variety of different means [15]. One means of altering immune responses is the production of superantigens. Superantigens [16,17] are either cellular proteins of viral origin [18,19] or bacterial exotoxins, such as Staphylococcus aureus enterotoxins (SEA, SEB, SEC-1-3, SED, SEE) [16]. Additionally, many studies have used superantigens as tools to examine T cell activation because they share several characteristics with conventional peptide antigen, like requiring MHC Class II presentation by APCs and stimulating cells through the TCR/CD3 complex, but also have the advantage of stimulating large numbers of T cells via their interactions with family-specific regions of TCR Vβ chains [16,20]. Because superantigens are microbial products, they may play a role in certain health settings. The bacterial exotoxins produce fever and lethal shock in experimental animals [21].

Superantigens have also been implicated in a number of human diseases such as streptococcal shock syndrome [22], acute rheumatic fever [23], and Kawasaki disease [24]. Superantigens are also commonly used to study peripheral tolerance (deletion and inactivation). Our own studies have contributed to this area, including the initial report that CD4 memory T cells are selectively non-responsive to SEB whereas naive cells respond vigorously to both conventional antigen and superantigen [12,25]. Additional studies, using a peptide-specific model, showed that if memory cells were exposed to SEB, they lost the ability to subsequently respond to cognate antigen [25]. Further, the induction of this “anergic” response is a consequence of impaired TCR proximal signaling and the activation of alternative signaling pathways. Altered signaling involved the hyperactivation of the src kinase Fyn which prompted a redistribution of the critical signaling molecule ZAP-70 away from the TCR complex and prevented downstream signaling [26,27]. Confirmation of the essential role that Fyn plays in SEB-induced anergy is indicated by the observation that memory CD4 T cells which lack Fyn, respond as vigorously as do naïve cells when exposed to SEB.

The functional consequences of memory cell anergy are unclear. However, it is likely that protection against infection would be impacted negatively. Given that superantigens can bind to large numbers of different TCRs and encompass peptide specificities beyond those present on the infecting pathogen, a host encounter with a pathogen that produces superantigens may have consequences with respect to pre-existing immunity against unrelated antigens. In the present study, we extend our previous observations on SEB-induced memory cell anergy to determine if there indeed is an impact on recall immune responses. Since a main function of CD4 T cells is to provide help for B cell Ab production, we investigated whether exposure to SEB would alter T-dependent Ab responses. Naive T cells primarily promote IgM secretion (i.e., primary response), even when the B cell is a memory cell [28]. In contrast, memory T cells help B cells to secrete both IgM and IgG antibodies (e.g., secondary response). We found that, indeed, vaccination of mice promoted an IgG Ab response upon secondary challenge. However, if the mice were exposed to SEB prior to challenge, only IgM Ab responses were observed. Hence, by targeting the memory T helper cells, recall humoral immunity was diminished. We conclude that exposure to this microbial superantigen negated the beneficial consequence of prior immunization to an unrelated antigen.

## Materials and Methods

### Animals

The BALB/c ByJ, DO11.10 [29], and DO11.10 × Fyn^−/−^ mice used in these experiments were bred and maintained at the Wadsworth Center Animal Core Facility under specific pathogen-free conditions. The majority of T cells in the DO11.10 and DO11.10 × Fyn^−/−^ mice are CD4^+^ cells which bear a TCR that recognizes a chicken ovalbumin-derived peptide, OVA(323-339) (hereafter referred to as OVAp), presented by I-A^d^ [29]. This TCR is encoded by transgenes encoding Vβ8.2/Vα13.1 chains and can be identified by the anti-clonotypic mAb, KJ1-26 [30]. Unless otherwise indicated, the experiments were performed using 8–12 week old mice. Both male and female mice were used in different experiments with no discernible differences in the results. All mice used in these studies were bred and maintained in accordance with the guidelines of the Committee on Care and Use of Laboratory Animals of the Institute of Laboratory Resources, National Research Council (Washington, DC). All experiments were approved by the Wadsworth Center IACUC.

### Reagents and antibodies

MAbs KJ1-26 (anti-DO11.10 clonotype) [30] and 23G2 (anti-CD45RB) [31], were prepared from the supernatants of hybridoma cell lines. Purchased Abs were: HRP-goat anti mouse IgG (Southern Biotech), HRP-goat anti mouse IgM (Southern Biotech), and Magnetic anti-goat mouse Ig microbeads, (Miltenyi Biotec). In addition, rabbit gamma globulin (RGG, Pel Freeze Biologicals, Rogers, AR) was purchased. Where indicated, RGG and BSA (Sigma) or whole chicken ovalbumin (OVA) (Sigma) were haptenated using 2,4,6-Trinitrobenzenesulfonic acid (J.T. Baker Chemical), as previously described. Chicken OVA peptide (OVA323-339) was synthesized and supplied by the Wadsworth Center Peptide Synthesis Core Facility. 5- (and-6)-carboxyfluorescein diacetate succinimidyl ester (CFSE) (Molecular Probes, Inc., Eugene, OR) and SEB (Toxin Technology), and ELISPOT kits (Becton Dickenson) were purchased.

### Preparation of cells

In all experiments, enriched populations of CD4^+^ T cells were prepared by negative selection procedures as previously described [32] and were 90–95% CD4^+^ and <3% sIg^+^ as determined by flow-cytometry. Naive and antigen-experienced cells, operationally referred to as memory cells, were then isolated based upon CD45RB expression using mAb 23G2, magnetic-goat anti-mouse Ig, and AutoMACS (Miltenyi Biotec) sorting to separate the CD45RB^hi^ (naive) and CD45RB^lo^ (memory) populations. Following separation, the sorting mAb was removed using a low pH buffer as described [33]. For proliferation analyses, the CD4 cells were labeled as previously described [25] with 5 mM CFSE prior to separation into naïve and memory populations.

### Immunizations, adoptive transfer, and analyses of DO11.10 cells

All immunizations were done i.p. Where indicated, primary immunizations were with 100 μg of TNP-RGG adsorbed to alum. Some mice were immunized with a mixture of 100 μg of TNP-RGG and 100 μg of OVAp adsorbed to alum. Additional injections included 25 μg SEB in PBS and/or 100 μg TNP-OVA adsorbed to alum. Adoptive transfers were done using the procedure described by Kearney et al [34]. Naive or memory DO11.10 CD4^+^ T cells (2 × 10^6^) were injected i.v. into BALB/c mice. After 24 h recipient mice were injected with PBS or SEB. In most experiments, after an additional 48 h the mice were immunized with TNP-OVA adsorbed to alum. For proliferation studies, 24 hours after cell transfer the mice were injected s.c. with either PBS, OVAp (150 μg) in PBS, or SEB (25 μg), with some mice also receiving one additional injection 24 h later with OVAp (150 μg) in PBS. For proliferation studies, 66 h after the initial injection, the axillary, brachial, and cervical lymph nodes were removed, pooled, and analyzed by flow cytometry using a FACSCalibur and CellQuest Software (Becton Dickenson). For enumeration of hapten (TNP)-specific antibody secreting cells, spleens were removed at the indicated times and assessed using an ELISPOT assay. Briefly, 96 well ELISPOT filter screen plates (Millipore) were coated with TNP BSA (0.4 μg/ml) overnight (4°C) and blocked with PBS/10% FBS. For background controls, similar plates were coated with BSA only. Unless otherwise indicated, mouse splenocytes from two mice per data point were pooled and the cells were suspended in tissue culture medium (RPMI-1640 medium supplemented with 10% FBS, 50 μM 2-mercaptoethanol, 100 U/ml penicillin, 100 μg/ml streptomycin, and 2 mM glutamine). Triplicate samples of serially diluted splenocytes were added to the filter plates and the cells were cultured for forty eight hours (37°C). Detection of bound Ab was done with either HRP-goat anti-mouse IgG or HRP-goat anti-mouse IgM, followed by visualization using an ELISPOT AEC kit (Becton, Dickinson). ELISPOTs were counted using a dissecting microscope. Statistical analysis was done using SigmaPlot (Systat Software, Inc).

## Results and Discussion

### Memory CD4 T cells do not proliferate in response to peptide antigen when first exposed to superantigen

In our previous studies we showed that exposure of memory, but not naïve CD4 T cells to SEB induces anergy. Consequently, these memory cells failed to proliferate or secrete cytokines when later exposed to a normally activating stimulus. The DO11.10 transgenic mouse strain is an excellent model for studying superantigen-induced anergy. The clonotypic TCR contains a Vβ8.2 chain [29] and readily interacts with SEB. The TCR binds to a well-defined peptide, OVAp, so that the response to both peptide and superantigen on the same cell can be studied. A clonotype-specific antibody, KJ1-26 [30], binds to the transgenic TCR and identifies cells from DO11.10 mice after adoptive transfer [34]. Finally, OVA-specific, KJ1-26^+^ memory T cells can be readily isolated from the lymphoid tissue of DO11.10 mice [33].

**Figure 1:**
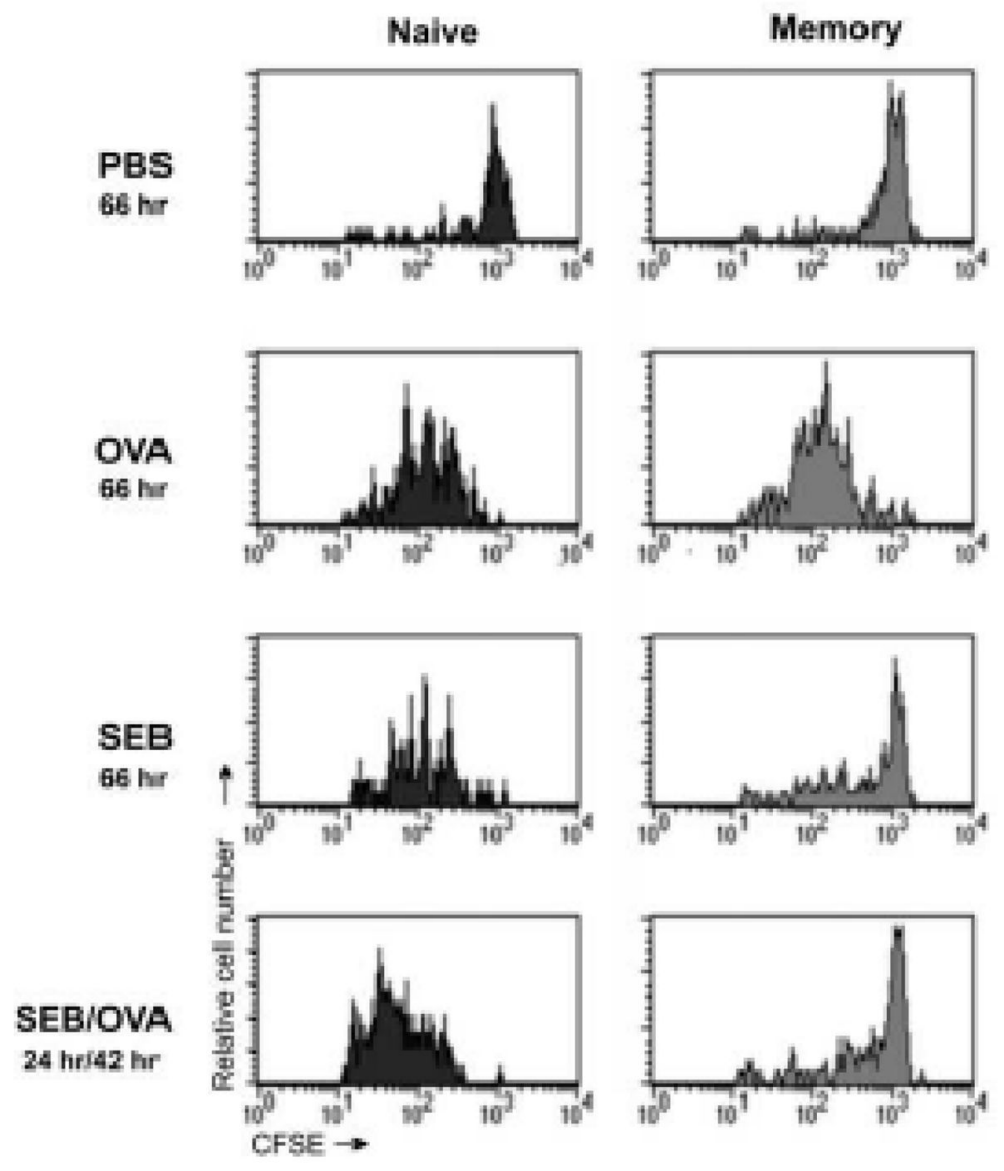
SEB induces proliferative anergy memory CD4 T cells *in vivo*. CFSE-labeled DO11.10 CD4^+^ naïve (left panels) and memory (right panels) T cells were injected into BALB/c mice. After 24 hrs of rest, the mice were immunized with the indicated agent, the times of exposure to each agent is indicated in the figure). In the bottom row, after an additional 24 h the SEB-injected mice were also immunized with OVA. At 66 h (42 h in the bottom row) after the last stimulus injection, lymph node cells were collected and stained with mAb KJ1-26, and proliferation was assessed. Cell proliferation was indicated by decreased fluorescence as determined by flow cytometry. Data are gated to show CFSE staining on viable KJ1-26^+^ cells.

While most of our earlier studies were done *in vitro* where we could quantify naïve and memory cell proliferation in response to SEB or OVA, we also showed that memory cell anergy could be induced *in vivo* [25]. In those experiments and as again shown in Figure 1, we used an adoptive transfer model [34] where naive or memory DO11.10 CD4 T cells were labeled with CFSE and were injected into Balb/c recipient mice. After the transfer, the recipient mice were injected with either OVAp or SEB, or with SEB followed 24 hr later by OVAp. Lymph nodes were isolated 3 days after the injection and proliferation of the donor KJ1-26^+^ donor cells was determined using flow cytometry. Figure 1 illustrates that unlike naïve cells, memory cells are not only refractory to stimulation with SEB but are also inactivated and lose the ability to proliferate in response to OVAp. These data were consistent with our *in vitro* studies that examined signaling mechanisms underlying memory cell anergy. Unlike the *in vitro* model, however, this experiment showed that proliferative anergy occurred in memory cells within the same physical environment that permitted simultaneous naïve cell activation. In this adoptive transfer model, recipient naïve CD4 T cells were present within the same lymph nodes as the donor memory cells. Given that some cytokines produced by naïve T cells, such as IL-2, permit memory CD4 cells to “escape” from anergy, it is noteworthy that memory cells still failed to proliferate in the mice that were injected with SEB. This suggests that normal exposure to superantigens through microbial infection might lead to selective targeting and inactivation of memory cells. Because the binding of superantigens is not related to peptide specificity, we speculated that gaps in memory cell repertoires to antigens unrelated to the infecting pathogen might develop. This might in turn lead to impaired immunity after vaccination and exposure to a superantigen. Hence, we tested whether SEB could block recall immune responses in immunized animals.

### SEB prevents exogenous (donor) memory CD4 T cells from helping B cells

**Figure 2:**
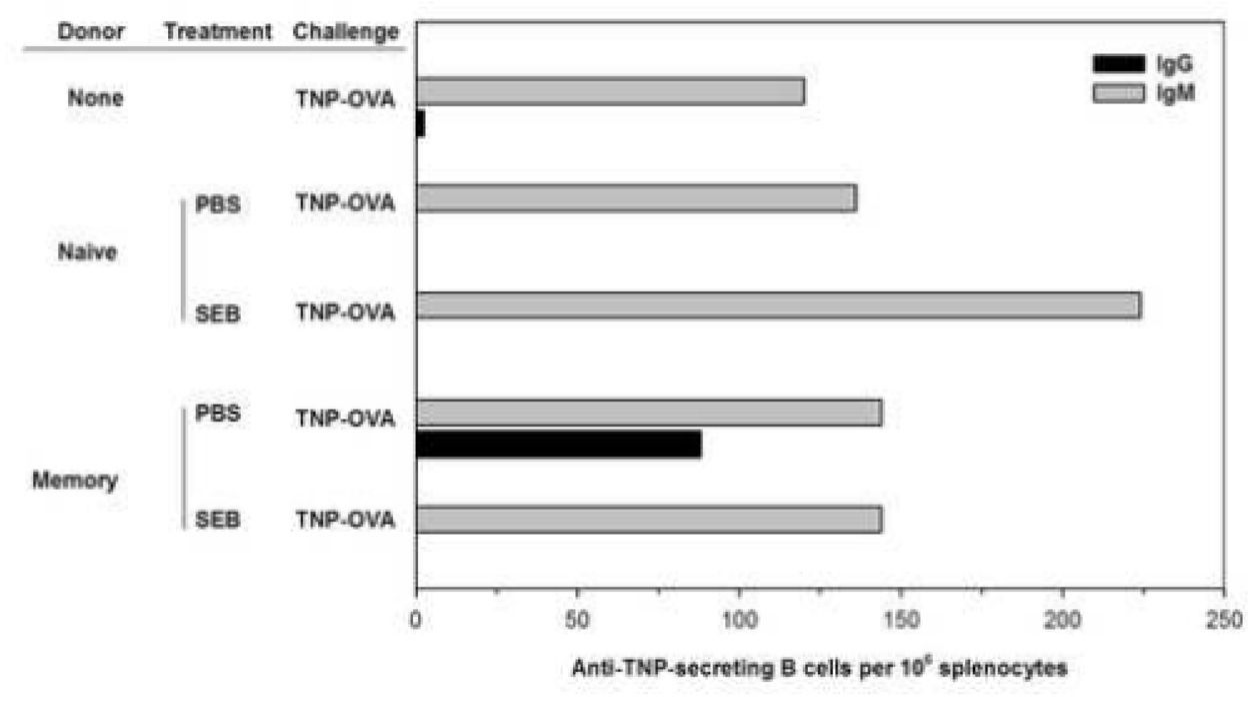
SEB interferes with exogenous helper memory CD4 T cell function to block B cell recall responses. Recipient BALB/c mice were primed with TNP-RGG adsorbed to alum. At 13 weeks, DO11.10 CD4^+^ naive and memory T cells were injected into the immune mice. 24 hours after adoptive transfer the mice were injected with either PBS or SEB, as indicated in the figure. After an additional 48 hours, all mice were challenged with an injection of TNP-OVA. Splenocytes were collected 5 days after immunization and IgM (gray) and IgG (black)-producing B cells were measured using a TNP-specific ELISPOT assay. Control mice were primed and also challenged but did not receive any donor cells. Data are from 2 pooled spleens per treatment and representative of three independent experiments.

A major helper function for CD4 T cells is to enable B cells to produce antibody. In order to determine if superantigen-induced anergy resulted in impaired recall responses, we examined the impact of SEB on antigen (hapten)-specific Ab responses. Vaccination primes both T cells and B cells so that “switched” antibody isotypes, predominantly IgG, are secreted upon subsequent exposure to antigen. Thus, a primary versus recall response should be indicated by the relative levels of specific IgM versus IgG, respectively. We first examined the impact of SEB on memory helper function using the DO11.10 adoptive transfer model to supply exogenous memory helper T cells. First, recipient BALB/c mice were immunized with TNP-RGG in order to generate TNP-specific memory B cells without promoting the generation of OVA-specific memory T cells. After 13 weeks the primary response had waned; circulating anti-TNP IgG could still be detected but no actively TNP-secreting B cells were found in the spleen using an ELISPOT assay (data not shown). As expected, if those mice were challenged with TNP-RGG, IgM and IgG anti-TNP-secreting B cells were detected (data not shown). For a recall antibody response, the booster immunization need not be the same hapten-carrier conjugate (TNP-RGG) used for priming, so long as the T cells were separately primed using the secondary carrier [35]. Hence, we hypothesized that OVA-specific memory cells would provide the appropriate help for memory B cells if the booster immunization was TNP-OVA. DO11.10 naïve or memory cells were transferred into the recipients at 13 weeks after immunization with TNP-RGG. After 24 h, the recipient mice were immunized with TNP-OVA and 5 days later the number of splenic B cells secreting anti-TNP were quantified using an isotype-specific ELISPOT assay (Figure 2).

Control mice that received no donor cells at all mounted a primary immune response; their spleens contained B cells that secreted IgM anti-TNP, but there was no detectable IgG anti-TNP. Hence, even though memory B cells were present, only endogenous naïve OVA-specific CD4 cells were available to help. Likewise, mice that received naïve DO11.10 donor cells made only a primary IgM Ab response. In contrast, the spleens of mice that received exogenous OVA-specific memory CD4 T cells contained both IgM and IgG-secreting B cells, indicative of a recall Ab response. We next determined if exposure to superantigens would alter the recall immune response. After adoptive transfer but 48 hours prior to the booster immunization with TNP-OVA, the recipient mice were injected with SEB. As indicated, mice that received naïve DO11.10 CD4 T cells were still able to mount an IgM anti-TNP response. Indeed, in most experiments, the numbers of IgM-secreting B cells were slightly higher than in control mice, suggesting that naïve cells were still effective at helping primary Ab responses. In contrast, SEB inhibited the resulting IgG Ab response in mice that received DO11.10 memory CD4 T cells. The IgM response, which we speculate was mediated by endogenous naïve T cells, was maintained. Hence, consistent with the observed proliferative anergy, SEB induced a selective functional impairment in memory CD4 T cells.

### Secondary humoral immune responses are impaired by SEB

**Figure 3:**
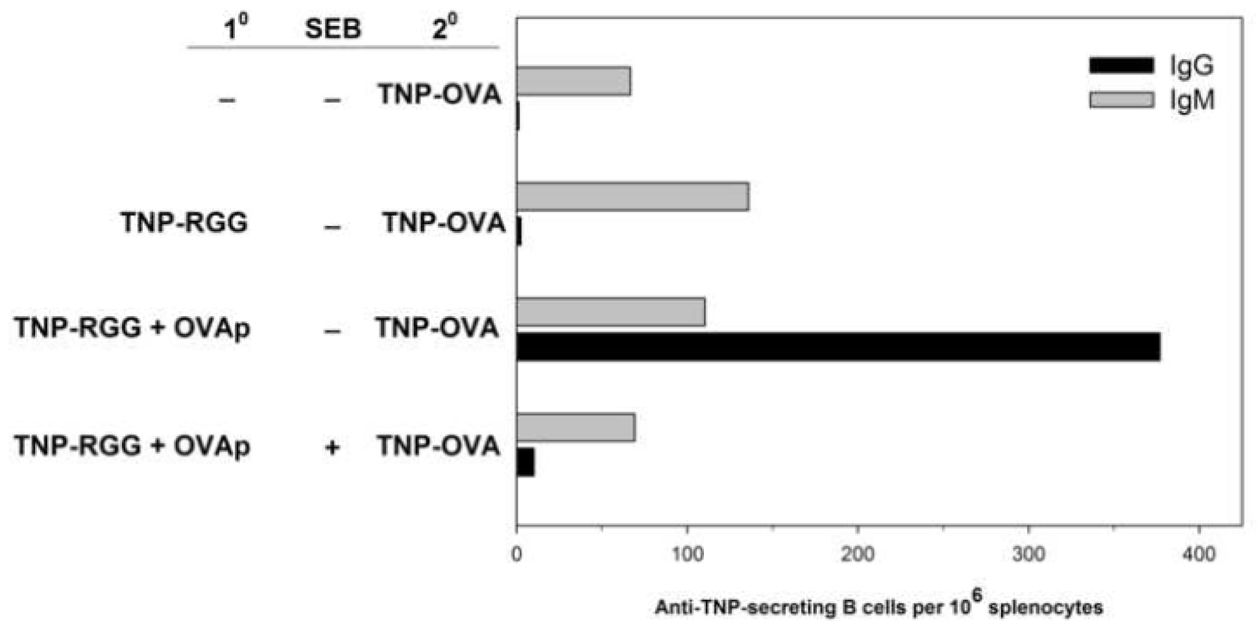
SEB interferes with secondary humoral responses in memory mice. BALB/c mice were immunized with a mixture of TNP-RGG plus OVAp adsorbed to alum (10). After 26 weeks, the immune “memory” mice were injected with either PBS or SEB, as indicated. After an additional 48 hours, the mice were challenged with an injection of TNP-OVA (20). Splenocytes were collected 5 days after immunization and IgM (gray) and IgG (black)-producing B cells were measured using a TNP-specific ELISPOT assay. The top two groups represent control mice that were either not primed or primed with only TNP-RGG to promote memory B cell development, respectively. Both control groups were challenged with TNP-OVA. Data are representative of two independent experiments.

We next wished to determine if SEB impacted recall immunity in normal, non-transgenic mice. Simple subunit vaccines, peptide vaccines, and epitope-enhanced vaccines can increase the precision of the T cell or B cell response (reviewed in [36]). However, that precision can lead to responses dominated by a limited number of responding lymphocyte clones. For example, when BALB/c mice are immunized with OVA323-339, the major responding T cell bears a Vβ8-containing TCR [30]. Hence, OVAp is a useful model for a peptide vaccine where the predominant responding T cell is also a target for SEB, allowing us to target a helper T cell response to antigen. Ideally, the vaccine would contain antigenic determinants for stimulating B cells and determinants for stimulating the helper T cells. However, because of its small size and to avoid interfering with the MHC or TCR binding portions of the OVA peptide, we chose to not directly haptenate the peptide for vaccination. As noted above, a linked hapten-carrier is not required to generate the memory B and T cells that will ultimately cooperate for secondary challenge. Rather, the T cells and the B cells may be initially primed by distinct antigens. Hence, BALB/c mice were immunized with a mixture containing TNP-RGG and OVA323-339 to generate memory B cells and memory CD4 T cells, respectively. To permit the primary response, including the presence of effector T cells [37], to wane, the mice were housed for at least 26 weeks. We did detect circulating IgG anti-TNP Ab, so as above, our measurements focused on activated B cells using an ELISPOT assay. We injected the immune mice with either SEB or PBS (control) and followed 48 h later with a booster immunization with a now linked hapten-carrier conjugate, TNP-OVA (whole ovalbumin). Although the whole ovalbumin should stimulate a broad number of T cells, we anticipated that the only memory CD4 T cells available to promote a memory B cell response (IgG secretion), were the cells stimulated by the original OVA323-339 epitope, and that any newly stimulated T cells, responding to additional OVA determinants, would promote only a primary (IgM) antibody response. Indeed, we found that mice that were immunized only once with TNP-OVA made only a primary Ab response (Figure 3). In addition, mice that were immunized with only TNP-RGG and then challenged with TNP-OVA, also exhibited only a primary humoral response (Figure 3). As these mice had only IgM TNP-secreting B cells, we concluded that there was a lack of OVA-specific memory helper cells. In contrast, if the mice were immunized with TNP-RGG^+^ OVAp, followed by a challenge with TNP-OVA, then both IgM and IgG TNP-specific ELISPOTS were found, suggesting that the initial priming, with OVA peptide, promoted development of memory helper T cells. Finally, if the mice were injected with SEB prior to challenge, then IgG production was reduced. Thus, OVA-specific Vβ8^+^ memory T cells were induced to provide help to B cells, and SEB caused their inactivation. Indeed, these mice appear as if they never received the initial vaccination.

### Impaired recall humoral immunity is long-lasting after exposure to SEB

**Figure 4:**
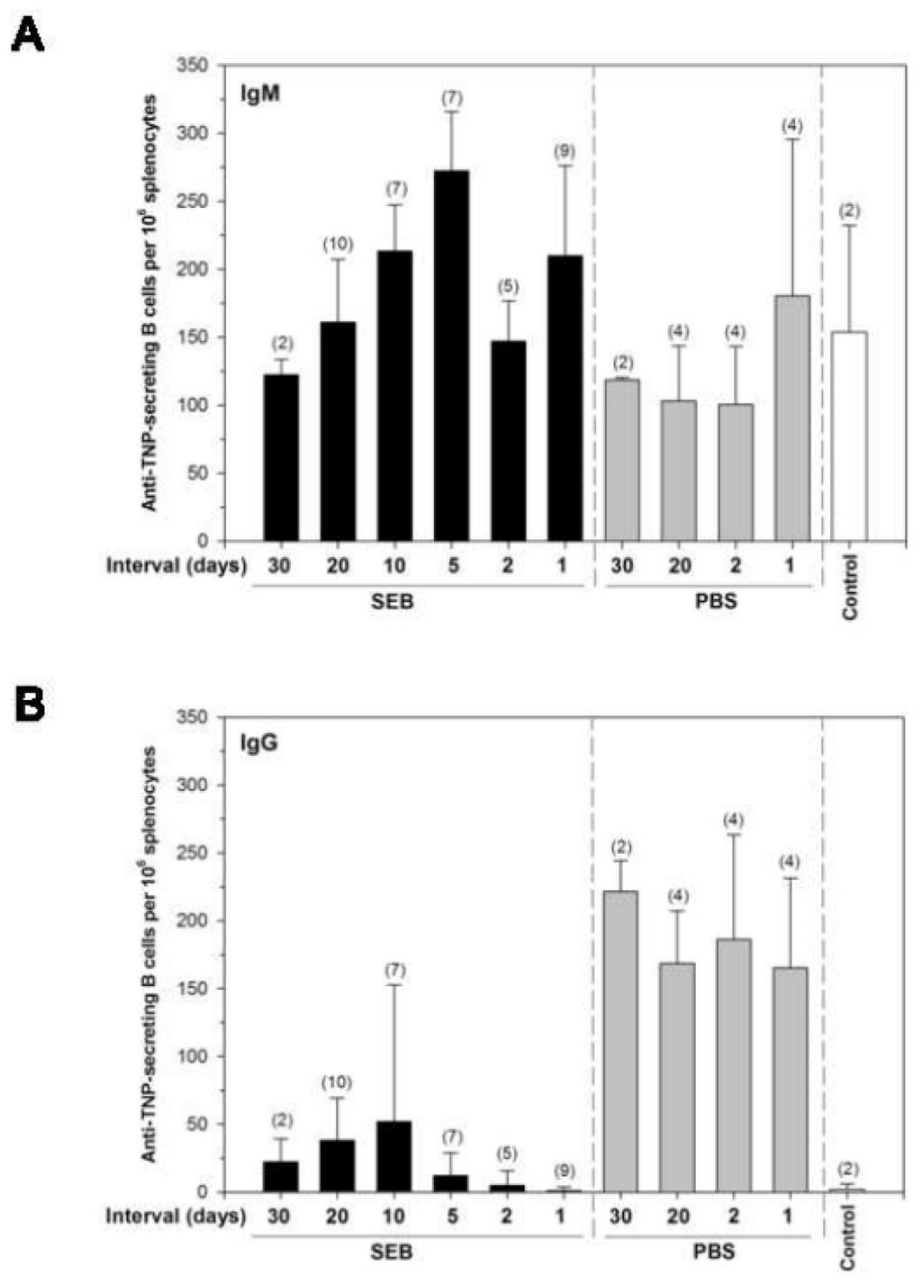
Antagonism of memory helper function by SEB is long-lasting. BALB/c mice were primed with a mixture of TNP-RGG plus OVAp adsorbed to alum. After 26 weeks, the memory mice were injected with either PBS or SEB. At the indicated time period (Interval), the mice were challenged with an injection of TNP-OVA. Splenocytes were collected 5 days after the challenge and (A) IgM and (B) IgG-producing B cells were measured using a TNP-specific ELISPOT assay. In both figures, the far right group represents control mice that were not primed but were challenged with TNP-OVA to initiate a primary Ab response. Each point represents data collected (based upon day 0 administration of SEB or PBS) from 3 independent experiments with the total number of mice indicated in parentheses. Data was analyzed by Two Way ANOVA. IgM values were not significant over time or between SEB and PBS treatment groups. IgG values were not significant over time but were significant between SEB and PBS treatment groups (p <0.001).

The preceding experiments demonstrated that a bacterial superantigen could selectively target CD4 memory cells and cause them to become unresponsive to stimulation with conventional antigen. We next wished to know whether this was a transient loss of protection or whether re-vaccination was necessary. To determine if the memory T cells would eventually recover helper function, we immunized mice with a mixture of TNP-RGG and OVAp and after 26 weeks we administered SEB or PBS. At increasing intervals we challenged the mice with TNP-OVA to see if an IgG anti-TNP response was elicited. Figure 4 shows composite data from a number of mice over three separate experiments. When we compared the number of IgM anti-TNP ELISPOTs from SEB-treated and control mice, we found that IgM was produced in all of the treatment groups (Figure 4A). The numbers of ELISPOTs varied between different experiments but the SEB-treatment groups usually contained as many, if not slightly more, IgM-secreting B cells. In contrast, there were few IgG-secreting B cells in the SEB-treated group as compared to the control mice, even at an interval of 30 days between the injection with SEB and the challenge with TNP-OVA (Figure 4B). These data suggest that the loss of recall immunity upon exposure to superantigens is long-lasting.

Several reports note that T cell anergy can be overcome by exogenous stimuli, such as IL-2 [38]. This is also the case with SEB-induced memory anergy, as *in vitro*, if memory cells are exposed to SEB along with exogenously added cytokines (IL-2, IL-1, IL-6), they will proliferate [12]. *In vivo*, it is unclear whether inflammation will restore recall helper function after exposure to superantigens and ongoing experiments are attempting to address this question. In part, reversing anergy would depend on whether the anergic memory cells are retained for a prolonged period. In previous experiments (Figure 1) [25], adoptively transferred DO11.10 cells were still detectable 4 d after exposure to SEB. Using flow cytometry we find very few donor cells beyond this time period, although the numbers that we do find 15 days after SEB administration are comparable to those found in mice that did not receive SEB (data not shown). Currently, we are attempting to detect DO11.10 donor cells by attaching mAb KJ1-26 to multiplex beads and detecting bound clonotypic TCR in cell lysates. In a pilot study, we determined that lysates of BALB/c spleen cells had only a background fluorescent signal but spiking them with lysates from DO11.10 mice resulted in an increasing signal that was proportional to the amount of DO11.10 cells. We did observe that pooled lysates from two recipient mice that had received DO11.10 memory cells and then SEB 15 days prior exhibited a positive fluorescent signal (data not shown). We are currently working to optimize this assay, but this preliminary result suggests that anergic memory cells may persist.

### Exogenous Fyn-deficient memory CD4 T cells support a recall humoral response after exposure to SEB

In our previous studies we have provided evidence that SEB directly tolerizes memory CD4 cells and prevents their function. Indirect mechanisms, such as suppression by regulatory T cells, which are also CD45RB^lo^, appear to have little role. For example, as we have previously discussed [14], we observe *in vitro* anergy even if the memory cells are isolated using surface markers other than CD45RB, such as CD44 or CD62L. Also, we find little evidence for activation of CD25^+^ positive cells or IL-10 secretion. Finally, we have shown a direct disruption of TCR proximal signaling when SEB is presented to memory T cells [27]. When SEB binds to the TCR on memory CD4 cells, negative signaling occurs through hyperstimulation of Fyn kinase [26,27]. This elevated activation in Fyn compromises the ability of ZAP-70 to migrate to the TCR-CD3 complex to be phosphorylated by the src kinase Lck and, consequently, downstream signaling is abrogated. Blocking Fyn activation prevents anergy. Hence, SEB directly tolerizes memory cells and prevents function. We suggest that *in vivo* SEB also directly signals memory cells, preventing activation and cytokine secretion and therefore eliminating B cell help responses. However, it remains possible that *in vivo* SEB indirectly affects memory cells through suppressive cytokine production or by activation of regulatory T cells. Previous studies have shown that SEB can activate Treg cells and that Treg cells play a role in dampening SEB-induced inflammation [39].

**Figure 5:**
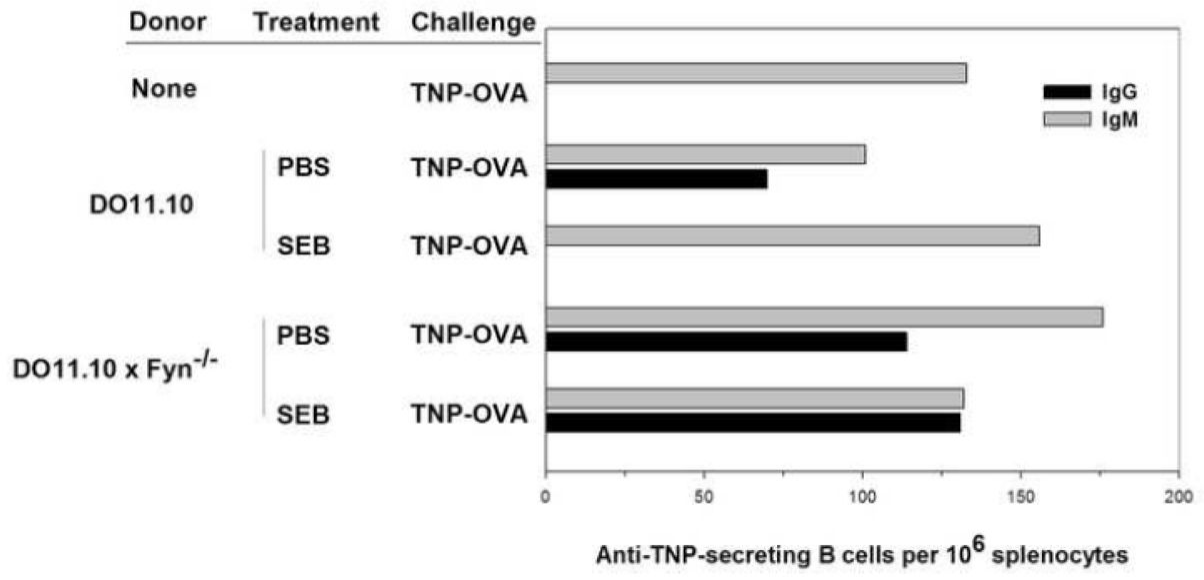
SEB-mediated antagonism of memory helper function is dependent upon Fyn kinase. Recipient BALB/c mice were primed with TNP-RGG adsorbed to alum. At 13 weeks, memory CD4^+^ T cells from donor DO11.10 or DO11.10 × Fyn^−/−^ mice CD4^+^ were injected into the immune mice and 24 hours after adoptive transfer the mice were injected with either PBS or SEB. After an additional 48 hours, the mice were challenged with an injection of TNP-OVA. Splenocytes were collected 5 days after immunization and IgM (gray) and IgG (black)-producing B cells were measured using a TNP-specific ELISPOT assay. Control mice were primed and then challenged but did not receive any donor cells. Data are representative of three independent experiments.

In order to address whether SEB directly inactivated memory cells, we adoptively transferred DO11.10 × Fyn^−/−^ memory CD4 T cells into TNP-RGG immunized BALB/c recipient mice followed by SEB administration and then challenge with TNP-OVA. *In vitro*, these memory cells respond well to initial stimulation with either OBAp or SEB; they do not become anergic. Further, the signaling defects that we previously identified to be associated with memory cell anergy are absent in the memory cells from Fyn ko mice. A substrate for Fyn is the adaptor molecule SAP [40–42]; SAP in T cells has been reported to be necessary for Ab responses to T-dependent antigens [43]. We were initially concerned that Fyn-deficient memory cells might not support an Ab response, independently of the impact of SEB. However, when we examined the recall humoral responses from the recipient mice, we found that the absence of Fyn led to a strong IgG anti-TNP response even when the mice were exposed to SEB before challenge (Figure 5). Hence, at least for memory cell helper responses, Fyn is not required. Whether SAP is required and is activated by a kinase other than Fyn is unclear at present. With regard to antagonism by SEB, these data suggest that impaired T cell helper function was a consequence of Fyn activation and most likely reflects a direct signaling effect by SEB on the memory T cell.

## Conclusion

In this study we have confirmed and extended our previous results showing that superantigens exert a strong and selective inhibitory effect on memory CD4 T cells. We show that memory cell anergy can occur *in vivo* and has consequences on immune function. Although we have used T-dependent B cell antibody production as a read-out, we anticipate that any CD4-mediated immune response would be similarly susceptible. The superantigen model has revealed a difference in regulation of memory cells versus naïve cells, initiated through TCR signaling. As we have previously suggested, distinct regulation paths may illustrate different self-recognition processes. However, we additionally feel that the data in this study may have implications with respect to pathogen modulation of host immunity. SEB is one of several superantigens which may be encountered in the context of infection. We suggest that such encounters may have negative consequences with respect to pre-existing immunity to antigen/pathogens unrelated to the source of the superantigen. Our future studies will address this hypothesis and will further examine the mechanisms by which responsiveness may be restored. Although superantigens can bind to a large number of different T cells, the overall responses to infection or to vaccines would cover multiple epitopes and diverse T cell families would be contained within the response. It is unlikely that impeding the response to one or a few epitopes, as might happen by exposure to a single superantigen, would severely impact recall protection. However, it may be that on those occasions where the T cell response is very narrow, due either the nature of the vaccine or the pathogen, the impact of eliminating an entire family, Vβ8-containing memory T cells for example, could have a significant effect.

## Abbreviations

SEB: Staphylococcal Enterotoxin B
TCR: T Cell Receptor for Antigen
Ab: Antibody
Ag: Antigen
OVA: Whole Ovalbumin
OVAp: OVA323-339
RGG: Rabbit Gamma Globulin
BSA: Bovine Serum Albumin
TNP: Trinitrophenyl
CFSE: 5-(and-6)-Carboxyfluorescein Diacetate Succinimidyl Ester
